# Topological analysis of protein co-abundance networks identifies novel host targets important for HCV infection and pathogenesis

**DOI:** 10.1186/1752-0509-6-28

**Published:** 2012-04-30

**Authors:** Jason E McDermott, Deborah L Diamond, Courtney Corley, Angela L Rasmussen, Michael G Katze, Katrina M Waters

**Affiliations:** 1 Computational Biology and Bioinformatics Group, Pacific Northwest National Laboratory, Richland, WA 99352, USA; 2 Department of Microbiology, University of Washington, Seattle, WA 98195, USA; 3 Knowledge Systems Pacific Northwest National Laboratory, Richland, WA 99352, USA

## Abstract

**Background:**

High-throughput methods for obtaining global measurements of transcript and protein levels in biological samples has provided a large amount of data for identification of 'target' genes and proteins of interest. These targets may be mediators of functional processes involved in disease and therefore represent key points of control for viruses and bacterial pathogens. Genes and proteins that are the most highly differentially regulated are generally considered to be the most important. We present topological analysis of co-abundance networks as an alternative to differential regulation for confident identification of target proteins from two related global proteomics studies of hepatitis C virus (HCV) infection.

**Results:**

We analyzed global proteomics data sets from a cell culture study of HCV infection and from a clinical study of liver biopsies from HCV-positive patients. Using lists of proteins known to be interaction partners with pathogen proteins we show that the most differentially regulated proteins in both data sets are indeed enriched in pathogen interactors. We then use these data sets to generate co-abundance networks that link proteins based on similar abundance patterns in time or across patients. Analysis of these co-abundance networks using a variety of network topology measures revealed that both degree and betweenness could be used to identify pathogen interactors with better accuracy than differential regulation alone, though betweenness provides the best discrimination. We found that though overall differential regulation was not correlated between the cell culture and liver biopsy data, network topology was conserved to an extent. Finally, we identified a set of proteins that has high betweenness topology in both networks including a protein that we have recently shown to be essential for HCV replication in cell culture.

**Conclusions:**

The results presented show that the network topology of protein co-abundance networks can be used to identify proteins important for viral replication. These proteins represent targets for further experimental investigation that will provide biological insight and potentially could be exploited for novel therapeutic approaches to combat HCV infection.

## Background

Recent advances in high-throughput methods for taking global measurements of transcript or protein levels from biological samples have driven the field of systems biology. A common application of such methods is to identify genes or proteins that are likely to be involved in the disease process being studied to direct further experimental investigation. These 'targets' are potential mediators of important aspects of the disease, or may be downstream responses to the disease process. Targets are generally identified from the most highly differentially expressed genes or proteins. However, this approach can overlook genes or proteins that are important, but may not be the most highly differentially regulated, such as transcription factors or other upstream mediators of critical processes [1]. In this study we extend our previous work showing that targets can be identified using network approaches based on global proteomics measurements [2]. We show that the differential regulation of a protein is an important factor in predicting biological significance, but that treating the data as a network and using topological measures allows for better prediction of biologically significant targets, provides better ranking of proteins, and allows extension by integrating other kinds of relationships, for example protein-protein interactions. Additionally we show that network topology of proteins is more conserved between experiments than is differential regulation. Our work provides a framework for network analysis of global proteomics data, and shows that this approach can identify biologically interesting targets.

Hepatitis C virus (HCV), a single-stranded positive RNA virus in the *Flaviviridae *family, is a major cause of liver disease in chronically infected individuals. Chronic infection causes inflammation and fibrosis of the liver and increases the chance of developing more serious hepatocellular carcinoma or cirrhosis in approximately 30% of infected individuals [3]. Current therapies have limited efficacy and numerous side effects [4] and a major challenge in translational hepatology research is the development of new approaches that target critical processes in the HCV life cycle and progression to disease state. Currently, study of HCV infection has been carried out in cell culture [2], on liver biopsy samples from infected patients [5], and in limited animal models [6], however similarities and differences between these different systems have not been extensively studied.

Previously we used global proteomics and lipidomics to show that HCV can reprogram cellular metabolism and bioenergetics in cell culture [2]. In order to identify possible targets through which HCV regulates metabolic reprogramming we constructed a correlation network based on global proteomics measurements of human hepatoma Huh7.5 cells responding to a time course of HCV infection [2]. We used the topology of the network, specifically proteins with high betweenness or bottlenecks, to identify biologically important proteins. Subsequently we showed that genetic silencing and pharmacological inhibition of one of these predicted targets, DCI, significantly inhibited processes critical for HCV infection [7]. These results showed the utility of network approaches to identify key components and interactions associated with HCV infection in cell culture experiments, but did not delineate how the approaches could be applied to provide the best results, nor if the approach would generalize to other proteomics data sets with very different experimental designs.

While our previous studies used network analysis to identify targets for further experimental investigation, they did not explore the generality and robustness of the approach. Though promising, the approach requires analyses of the parameters used for network generation and target identification, analysis of topological measures beyond betweenness centrality, and application to other similar data sets. Only by exploring these aspects can the significance and applicability of the approach be established. The current study had two principal aims. The first was to evaluate these factors for network-based target identification from proteomics data and to compare this approach with an existing method for identification of important proteins from global proteomics data, differential regulation. This is particularly important work because proteomics technology has recently reached a point where it is possible to generate studies with multiple global proteomics datasets of the system being studied under different conditions and there have been very few reports describing use of proteomics data in network inference approaches. The second aim was to compare network-based analysis of proteomics from HCV infection in cell culture experiments with similar networks generated from liver biopsy samples to identify common targets that have potential translational impact. These aims represent an important and significant advance over our previous work because we systematically compare our network topology approach with traditional approaches to target identification, characterize the impact of network inference parameters on our results, and compare the results obtained in our cell culture studies with those obtained from clinically relevant patient-derived samples.

In order to further explore the identification of novel, translationally relevant pathways and important proteins involved in HCV infection and liver disease progression, we first analyzed the topology of networks inferred from the time course study of HCV infection in cultured Huh7.5 cells with an emphasis on now evaluating the ability of various topological measures to predict proteins known to be targeted by pathogens in general and HCV proteins specifically. As described above, we further integrated protein-protein interaction data in the networks and showed that the integrated networks provide improved discrimination of important proteins using network topology. An important observation from this analysis was that network topology provided better discrimination of important proteins than differential regulation. We then reanalyzed proteomics data from a previous cross-sectional study of HCV infected patients [5] using the same approaches. We obtained similar trends in this analysis for identification of important proteins *in vivo*. In addition we found a number of proteins that share important topological roles in networks inferred from both the *in vitro *system and the *in vivo *clinical samples. We conclude that considering proteomic data as networks highlight important *in vivo *proteins from examination of *in vitro *systems thus, providing valuable insight into translationally relevant disease processes.

## Methods

### Datasets

We used two datasets in this study that have been previously described. The first is from the Huh7.5 human hepatoma cell line infected with a chimeric HCV genotype 2a virus, J6/JFH-1 [2]. Cells were inoculated with HCV or UV-inactivated virus and samples taken at 24, 48, 72, and 96 hours post-infection. The samples were analyzed by liquid chromatography-mass spectrometry (LC-MS) using the accurate mass-and-time tag (AMT) approach in combination with trypsin-catalyzed ^16^O/^18^O labeling for quantitation [8,9]. Briefly, peptides from time-matched mocks were individually labeled with ^18^O and spiked at equal amounts into the appropriate HCV or UV-HCV-inoculated sample. The corresponding ^18^O/^16^O intensity data from multiple observations of the same protein were then rolled up to compute a final protein abundance ratio for all proteins identified in a given sample and, to identify those proteins exhibiting statistically significant (*p *< 0.05) changes in abundance compared to the control sample [2].

The second dataset used was from HCV-infected liver tissues from 15 patients at different stages of fibrosis [5]. This study also employed stable isotope ^16^O/^18^O trypsin catalyzed labeling in combination with the AMT tag approach for protein quantitation. In this case, proteins exhibiting statistically significant (by ANOVA on fibrosis stage groups [5]; *p *< 0.05) changes in abundance were determined relative to a control sample consisting of peptides generated from a pool of 8 HCV-positive patients with minimal liver disease as previously described [5].

The list of proteins known to be physically targeted by pathogens (interactors) was taken from supplemental material in [10]. A list of proteins identified in a two-hybrid screen as interacting with HCV proteins was obtained from supplemental material in [11]. Mouse homologs were obtained from the Mouse Genome Database (MGI) [12]. A list of human genes that exhibit positive selection was obtained from the Human PAML Browser [13] available at http://mendel.gene.cwru.edu/adamslab/cgi-bin/paml/pbrowser.py using a significance threshold of *p *< 0.01. Protein-protein interactions were obtained from http://cytoscape.wodaklab.org/wiki/Data_Sets.

### Proteomics data filtering and network construction

To construct association networks from proteomics data we used a multi-step procedure that involved three parameters for network construction as follows:

1. Proteomics data was filtered for significance.

2. Data was converted to a ratio versus control conditions.

3. A filter was applied to remove differential abundance ratios below a threshold (**abundance filter**).

4. Correlation values were calculated between present values for all pairs of proteins.

5. A filter was applied to remove correlation values with a number of comparisons below a threshold (**correspondence filter**).

6. A filter was applied to remove correlation values below a threshold (**correlation filter**).

Significance filtering and ratio calculation are described above and in the original papers [2,5]. The **abundance filter **(step 3) replaces all values with ratios below the threshold with missing values in the vector of abundance ratios for each protein. Correlation is calculated as the Pearson correlation coefficient for all pairwise complete observations (steps 4). Correlation values with a low number of comparisons are removed (set to 0) according to the **correspondence filter**, where a single comparison is counted if abundance ratios are observed for the same condition for the pair of proteins being considered. Finally, the **correlation filter **is used to generate a final adjacency matrix, which is then treated as a network for topological analysis. Previously, the impact of the choice of similarity threshold on construction of coexpression networks has been investigated [14,15]. However, in this study we have chosen reasonable values for these parameters by evaluating the topological enrichment of the resulting network in proteins known to be targeted by pathogens (see Results). For topological analysis (below) we varied parameters for the three filters listed here to generate multiple different networks.

### Topological analysis

Topological analysis of networks was performed using in-house scripts in the statistical language R http://www.r-project.org/ that utilize the igraph R library http://igraph.sourceforge.net/. We provide our code in (Additional file 1). Advanced topological analysis was performed using the network analysis software UCINET 6.0 http://www.analytictech.com/ucinet/. Examples of advanced topology metrics are reachability [16], Katz influence [17], and Bonacich power centrality [18]. The clustering coefficient, and degree, closeness and betweenness centrality metrics are defined as below [19,20].

*Degree Centrality *- this is a metric of the connectedness of a node. It is simply a count of the number of edges that attach to a node. For a graph G with *n *vertices, edges *e *the degree centrality *C_D_(v) *for vertex *v *is:

$$C_{D}\left(v\right)=\frac{\deg\left(v\right)}{n-1}$$

Generally, degree centrality is the fraction of edges for a particular protein out of all possible interactions for that protein in the network.

*Closeness Centrality *- a metric defined as the average shortest paths or geodesic distance from vertex *v *and all reachable vertices (*t *∈ *V*\*v*) where *n *is the number of proteins in the graph and:

$$C_{C}\left(v\right)=\frac{\sum_{t\in V\backslash v}dist\left(v,t\right)}{n-1}$$

Generally, closeness is the mean distance between a protein and all other proteins in the network.

*Betweenness Centrality *- a metric that measures how often paths between nodes must traverse a given node (i.e., influence). Specifically, a vertex *v *is central if it lies between other vertices on the shortest path between them. That is, the vertex is "between" many others. Where *g*_jk _is the number of paths linking vertex *j *and vertex *k *is:

$$C_{B}\left(v\right)=\sum_{j\neq k\neq v\in V}\frac{g_{jk}\left(v\right)}{g_{jk}}$$

Generally, betweenness is the number of shortest paths between all pairs of proteins in the network that pass through a specific node.

*Eigenvector Centrality - *is a metric that can be thought of as recursive degree centrality [18]. This centrality can be calculated by the algorithm that starts by assigning 1 to all of the nodes, then the scores of each node are recomputed as weighted sum of centralities of all nodes in a node's neighborhood (N):

$$v_{n}=\sum_{j\in N}x_{ij}v_{j}$$

then normalize the centrality by the largest value and repeat until the values converge. Generally, a protein with high eigenvector centrality is connected to other proteins who themselves are connected to many other proteins.

*PageRank Centrality -*is a type of eigenvector centrality and measures the importance of a node by assuming links from more central nodes contribute more to its ranking than less central nodes [15]. Let *d *be a damping factor (usually 0.85), *n *be the index to the node of interest, *v_n _*be the node, M(*v*_i_) be the set of nodes linking to *v*_n _and L(*v*_j_) be out-link counts from node *v*_j_:

$$PR_{v_{n}}=\frac{1-d}{N}+d\sum_{vj\in M\left(v_{n}\right)}\frac{PR\left(v_{j}\right)}{L\left(v_{j}\right)}$$

Generally, page rank is the importance of a protein in the network.

*Clustering coefficient *- a statistic describing the overlap in the network topology. The clustering coefficient *CC_v _*is the probability that any two nodes are linked together if they have a neighbor in common. Let vertex *j *and vertex *t *be in the neighborhood of vertex *v *and *e(j, t) *is an edge the graph. For undirected graphs, the clustering coefficient of vertex *v *is defined as:

$$CC_{v}=\frac{2\left|\left\{e\left(j,t\right)\right\}\right|}{k_{v}\left(k_{v}-1\right)}$$

Generally, the clustering coefficient is defined as the percentage of neighboring proteins that interact with each other

### Functional enrichment analysis

Enrichment of a population (for example, the top 20% of proteins in terms of betweenness) for a particular functional label was calculated using the hypergeometric test. Functional labels were defined by the list of pathogen or HCV targets, or positively selected genes. In all cases the background for significance was the total list of proteins determined to be significantly differentially regulated not including the population in question. Significance levels are indicated in the text but in general a *p*-value of 0.05 or below was considered to be significant. Where indicated, multiple hypothesis correction was applied to *p*-values using the Bonferroni correction.

## Results and discussion

### Highly abundant proteins are more likely to be targeted by pathogens

**Figure 1:**
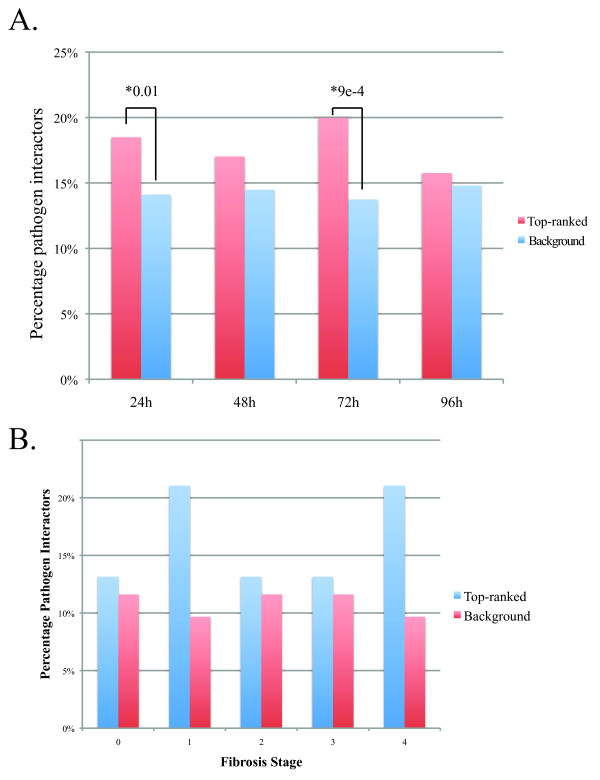
**Highly differentially regulated proteins are preferentially targeted by pathogens and HCV**. **A**. Pathogen interactors are enriched in differentially expressed proteins from cell culture experiments. The percentage of known pathogen interaction targets in general is shown for the top 20% of proteins ranked by differential regulation overall at each time point post-infection (red bars) versus background (blue bars). Statistically significant enrichment is indicated by asterisks with *p*-values less than 0.05 by Fisher's exact test. **B**. Pathogen interactors are enriched in proteins differentially expressed in patients with severe fibrosis. The percentage of pathogen targets in the top 20% of differentially regulated proteins is shown (red bars) versus the percentage in the other proteins (blue bars). None of these comparisons was significant by Fisher's exact test.

We first investigated whether differential abundance relative to control (differential regulation) could be used to identify proteins with more importance for infection. Importance was assessed by testing for enrichment in proteins known to be physical interaction partners with proteins from multiple pathogens [10] and also for those specific to HCV [11]. Using the proteomics data from a time course infection of human hepatoma cells (Huh7.5) previously described [2], a total of 2378 observed significant (*p*-value < 0.05) proteins were ranked based on the change in differential regulation relative to uninfected cells at each time point (24, 48, 72, or 96 hours post-infection). We assessed the enrichment of the top 20% (475 proteins) in the pathogen interactor or HCV interactor lists (Additional file 2: Table S1) using the Fisher's exact test. Figure 1A shows that highly differentially regulated proteins are more likely to be targets of pathogen interactions. Based on previous observations we also assessed the tendency of more differentially regulated proteins to exhibit positive evolutionary selection. However, we found neither group to be more subject to positive evolutionary selection (data not shown). Additional file 2: Table S1 provides details of this analysis. This shows that pathogens target more differentially regulated proteins in general, and that proteins differentially regulated early in infection are preferentially targeted by HCV.

Analysis of patient samples should provide a more direct assessment of the validity of potential clinical targets, compared to *in vitro *experimental models. We examined data from a previous study analyzing liver biopsy samples from 15 patients at five stages of fibrosis [5] to compare our observations in cell culture. We compared proteins from the 15 infected patients against a pool of 8 HCV-positive control patient samples. Significance was assessed using ANOVA resulting in 210 significantly changing proteins, and 193 of these proteins were associated with a gene symbol and could be used for enrichment calculations with the pathogen interactor lists. Our results are presented in Figure 1B and show that the most differentially regulated proteins (top 20%) are enriched in proteins that are pathogen interactors in fibrosis stages 1 and 4, however, these differences are not statistically significant due to the smaller number of significant proteins identified.

### Network topology identification of HCV targeted proteins

**Figure 2:**
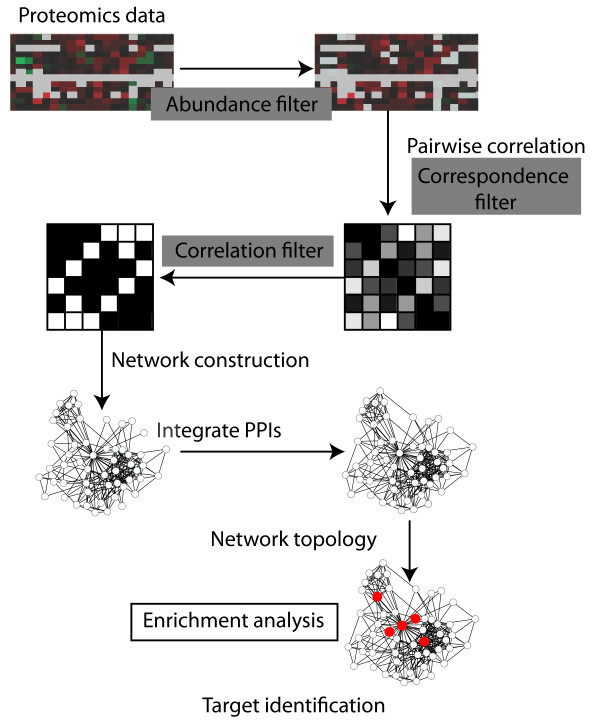
**Overview of network inference and topological target identification from proteomics data**. The filters used in proteomics processing are shown in grey boxes (see text).

Previously we reported that topological analysis of networks inferred from proteomics data could be used to identify proteins that are targets of interaction by HCV proteins [2]. Additionally, another paper has described the importance of protein-protein interaction network topology in identification of interaction targets of pathogens [10] and a recent study of the interaction networks surrounding HCV-specific interaction targets also highlighted network topology [21]. We wanted to determine if our previous results could be improved upon by optimizing the parameters in network inference and employing different topological measures. Accordingly we inferred protein association networks using different parameters (see Methods). Briefly networks were constructed by filtering differential regulation data using an abundance filter, ***j***, and constructing a correlation matrix where only pairs of proteins with ***k ***or more valid comparisons were retained (correspondence filter). A missing value, where no protein is observed, in the abundance profile of either protein considered, does not count toward valid comparisons. The correlations were then filtered to retain only correlation values at or above a threshold of ***r ***(correlation filter). Topological measures (degree, betweenness centrality, closeness and clustering coefficient) were calculated for all proteins in the resulting networks. The top proteins as ranked by each measure, were assessed by statistical enrichment in either known pathogen interactors [10] or specific HCV interacting proteins [11] versus the remainder of proteins in the network. Figure 2 shows an overview of this process.

**Figure 3:**
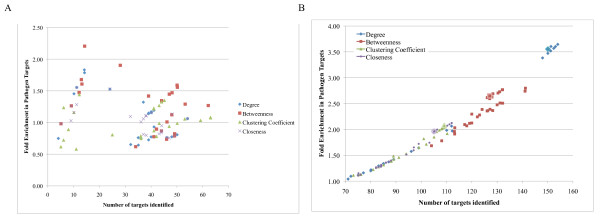
**Network analysis of global proteomic data from HCV infection of Huh-7.5 cells allows identification of targets**. We constructed association networks from proteomics alone **(A) **or with added protein-protein interactions **(B) **as described in the text, varying parameters as indicated in (Additional file 3: Table S2). The top 20% of proteins ranked by the topological degree (blue diamonds), betweenness (red squares), clustering coefficient (green diamonds), and closeness (purple x's) were evaluated for their enrichment in proteins that are known interaction partners of pathogen proteins. Fold enrichment (Y axis) is calculated as the percentage of pathogen interactors in the group divided by the percentage not in the group. In panel B the circled points indicate the values for the PPI network alone. Statistical significance is indicated in (Additional file 3: Table S2).

We present the results of this analysis in Figure 3A and show the number of pathogen interactors 'identified' (that is, in the selected group) on the X axis, versus the fold-enrichment in pathogen interactors (percentage of pathogen interactors in the selected group divided by the percentage of pathogen interactors in the remainder of the network) on the Y axis. Selected groups, the top 20% of proteins ranked by degree (diamonds), betweenness (squares), clustering coefficient (triangles), and closeness (circles) are indicated for varying network inference parameters. Each point on this graph represents a distinct set of parameters for network generation and a complete table of results including statistical significance for each enrichment value is provided in (Additional file 3: Table S2). These results show that all four topological measures that betweenness performs better than the other three measures with the highest enrichment and the greatest number of interactors covered (indicated by the point furthest along the diagonal). To summarize this observation across all networks tested we summed the numbers of pathogen targets in each group (top 20% of proteins ranked by each topological property) versus the number found in the remainder of the network across all networks and evaluated the significance of the enrichment found using Fisher's exact test. These results are included in Table 1 and show that enrichment in pathogen targets is significant (*p*-value < 0.01) for betweenness and clustering coefficient, but not for degree or closeness, though there are a number of individual networks that show statistical enrichment in these topological properties.

**Table 1:** Networks inferred from proteomics show significant enrichment in pathogen targets across many network inference parameters

	Measure	Group	Background	*p*-value	Fold-enrichment
^**a**^**CoA**	Degree	18%	18%	1.6E-01	1.0
	
	Betweenness	20%	17%	1.4E-07	1.2
	
	Clustering Coefficient	19%	17%	7.1E-03	1.1
	
	Closeness	19%	17%	1.2E-02	1.1

**CoA + PPI**	Degree	35%	14%	2.2E-16	2.5
	
	Betweenness	46%	12%	2.2E-16	3.8
	
	Clustering Coefficient	31%	15%	2.2E-16	3.8
	
	Closeness	32%	15%	2.2E-16	2.1

^a^CoA, co-abundance networks; CoA + PPI, co-abundance with protein-protein interactions

Following our previous approach we combined each network with experimentally determined protein-protein interactions (PPIs) between observed proteins. In this process known PPIs between proteins already in the co-abundance network are added as new edges to the network. The results of this analysis are shown in Figure 3B. Open symbols show the enrichment in the PPI network alone. These results show that the inferred protein association relationships can improve target discrimination using each topological measure except clustering coefficient, but that the best discrimination is provided by degree followed by betweenness. The specificity and sensitivity of both the degree and betweenness approaches are significantly better than either differential regulation or the inferred networks without PPIs. Table 1 provides a summary enrichment across all networks (full results in Additional file 3: Table S2). These results show that all four topological measures provide highly significant (*p*-value < 1e-16) enrichment in pathogen targets, with betweenness and clustering coefficient displaying highest enrichment, thereby demonstrating the added value of incorporating PPI data into inferred networks for a generalizable approach to identify target regulatory nodes within networks.

**Figure 4:**
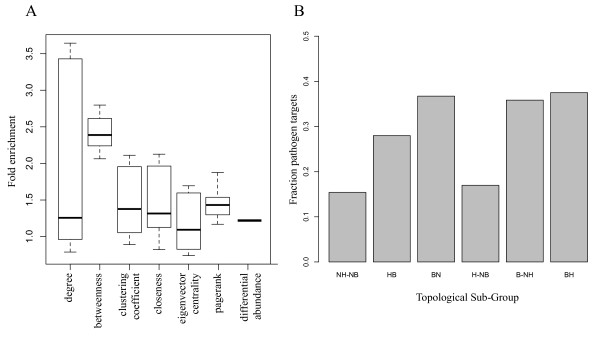
**A. Comparison of topological measures in identification of important proteins**. Networks were inferred as described in the text, varying parameters (see Additional file 3: Table S2), and topological measures calculated (X axis). The enrichment of known pathogen interactors in the top 20% of proteins ranked by each measure was calculated and is shown as the percentage enrichment in the group divided by the enrichment in background. The boxes represent the 25^th ^and 75^th ^percentiles, the bold bar represents the mean, and the dashed bars are at 1.5 times the interquartile range. The enrichment given by differential abundance is shown as a comparison. This figure shows that degree and betweeness perform much better than other, more sophisticated, measures of importance for these networks. **B**. Comparison of the enrichment of topological sub-types. For the cell culture co-abundance network integrated with PPIs we assessed statistical enrichment in pathogen targets for the following sub-types of topology: NH-NH, non-hub non-bottleneck nodes; HB, hubs; BN, bottlenecks; H-NB, hub non-bottlenecks; B-NH, bottleneck non-hubs; BH, bottleneck hubs. These sub-types were determined as the overlap of the top 20% of nodes ranked by degree (hubs) or betweenness (bottlenecks). The analysis shows that betweenness is the primary driver of importance in these networks similar to observations in directed regulatory networks.

There have been a large number of different topological measures developed for various purposes. We were interested in comparing more sophisticated measures with those considered in Figure 2 to see if any would provide better identification of interesting target proteins. We assessed the enrichment of the top 20% of proteins ranked by each measure in pathogen interactors for all the networks generated above. We examined two popular topological measures, eigenvector centrality [18] and pagerank [22]. Both of these measures were developed to provide some measure of the importance of the node in a network. With an enrichment score of 2.4, betweenness performs much better than any other topology metric (all others below 1.5) at identification of important proteins defined by prior knowledge, at least for this kind of network (Figure 4A).

It is well known that some topological measures display varying degrees of overlap; for example, proteins may have both high betweenness and high degree and thus be bottleneck-hubs [23]. We were interested in assessing the relationships between topological measures in our integrated network using Spearman rank correlation (to account for differences in the distributions of these measures). The results are presented in Additional file 4: Figure S1 and show that degree and closeness are highly correlated in all of the networks examined, while betweenness was slightly less correlated with these two and clustering coefficient was the least correlated with the other measures. Eigenvector centrality was highly correlated with degree and closeness, but pagerank was not as correlated with the other measures.

Examining degree and betweenness, the most used metrics for biological networks, we found that of 343 bottlenecks and hubs (the top 20% of proteins as ranked by betweenness and degree, respectively), 184 (53%) were shared, reflecting the moderate correlation between degree and betweenness and the fact that they aren't capturing the same characteristics of the networks. To examine this overlap further, we assessed whether the enrichment of bottlenecks in pathogen targets was dependent on their hub status within the integrated cell network. In Figure 4B we show the results from a topological subgroup analysis (similar to [23]) showing enrichment in pathogen targets for various overlapping groups. Interestingly, these results show that betweenness alone contributes more to importance than does degree, since the hub and hub-nonbottleneck groups are less enriched than the bottleneck, hub-bottleneck, or nonhub-bottleneck groups. Similar results were observed in the previous study by Yu, *et al. *[23] for regulatory networks, but not for PPI networks, indicating that our inferred networks combined with PPIs maintain the properties of regulatory networks and are less similar to PPI networks.

### Functional characterization of topologically-defined targets

Given their enrichment in pathogen interactors, we hypothesized that proteins with high betweenness might be involved in similar functions. We therefore investigated this in the network with the best enrichment from the analysis above. This was a network that was given by an abundance filter of 0, a correspondence filter of 4, and a correlation filter of 0.9 (see Methods). We then assessed the top 20% of the proteins in this network ranked by betweenness for functional enrichment in gene ontology categories. Despite the fact that the bottleneck proteins from this network were the most enriched for pathogen interactors as well as for HCV-specific interactors, we found no significant enrichment in any functional categories, relative to the other proteins in the network. This indicates that these proteins are united by their importance to the replication of HCV, but span diverse functional categories.

### Application of network analysis to clinical proteomics data

**Figure 5:**
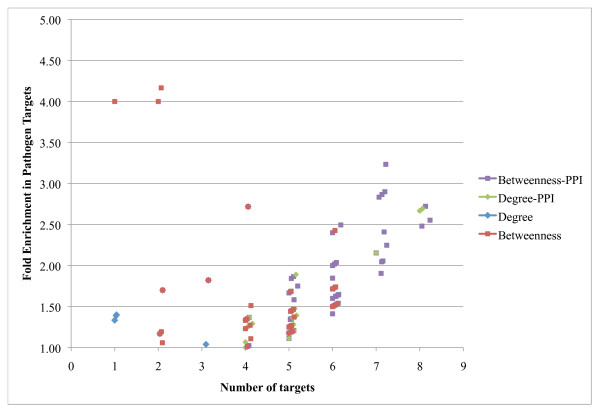
**Network analysis of global proteomic data from liver samples of HCV-positive patients**. We constructed association networks from proteomics as described in the text, varying parameters as indicated in (Additional file 5: Table S3). The top 20% of proteins ranked by the topological degree (blue diamonds), betweenness (red squares), clustering coefficient (green diamonds), and closeness (purple circles) were evaluated for their enrichment in proteins that are pathogen interactors. Fold enrichment (Y axis) is calculated as the percentage of pathogen interactors in the group divided by the percentage not in the group. Statistical significance is indicated in (Additional file 5: Table S3).

We were interested in whether this approach to target identification from proteomics data would generalize to other data sets, and so used proteomic data from the study of HCV-positive patients with various stages of fibrosis of the liver [5], mentioned above. We applied a similar network inference approach to the liver biopsy data, inferring networks using different ranges of parameters than for the cell culture data. The results of this analysis are shown in Figure 5. We chose to focus on just the degree and betweenness topology measures for the figure, and show enrichment in both the inferred networks and the networks combined with PPIs. Though the numbers are much lower in these networks, they are comparable to the results obtained in the cell culture data and the points of highest enrichment are statistically significant (see Additional file 5: Table S3 for details). Additionally, an overall significance calculated from networks using all parameters shows that betweenness is the only measure significantly enriched in pathogen targets, and this overall enrichment is only significant (*p*-value 1e-9) in the coabundance networks integrated with PPIs (Additional file 6: Table S4). However, we note that many individual networks with different parameter sets are significantly enriched. These results show that our approach can work similarly on data from time course experiments in cell culture and from clinical samples from multiple patients. It also indicates that betweenness is the only topological measure that is robust across the two different datasets.

### Network topology is more conserved than differential regulation

The Huh7.5 cell culture system has been extensively used as a model for HCV infection [2,24-26]. However, it is unclear at the molecular level how common patterns of expression and regulation might be in terms of HCV pathogenesis and disease progression. To examine this we used two approaches: examining the correspondence in differential regulation between the two datasets and examining the correspondence of topological characteristics in the networks described. For network comparison we chose to compare the networks with the best topological enrichment of pathogen targets, as described for the cell culture network above. For the liver biopsy derived network we chose a correspondence filter of 5, abundance filter of 2, correlation filter of 0.8, with integrated PPIs (see Additional file 3: Table S2). The filters used were different than those used for the Huh7.5-derived network because the structure of the underlying data sets were different. The liver biopsy data contains data from 15 patients and thus the number of corresponding data points used is more (5 versus 4) and the abundance filter is related to the overall range of differential abundance so the difference here (2 versus 0) reflects the larger variance observed in the patient samples. These differences highlight the fact that some care must be used when applying these methods to different kinds of datasets.

We first compared differential regulation in the 148 proteins that were observed in both the cell culture samples and the liver biopsy samples. Differential regulation ratios for infected samples were averaged per protein across all time points or patients. This process provides a reasonable estimate of the overall differential regulation for a protein in each experiment. We then compared the abundance ratios for proteins identified in both datasets using Spearman rank correlation. The two lists displayed no correlation (R = -0.05) indicating that the overall level of differential regulation in the cell culture system is not a good indicator of differential regulation in the liver biopsy samples. To ensure that this result did not reflect the use of averaged differential abundance across patients and time points that could mask true correlation between the two groups, we also calculated the Spearman rank correlation between the differential abundance ratios for all fibrosis stages from patients versus all time points. These results confirmed our findings; the mean correlation in differential abundance between different stages of fibrosis was 0.63, and between different time points was 0.24, whereas the mean correlation between the two sets was 0.03. The maximum correlation between any two fibrosis stages was 0.73, and between any two time points was 0.74, whereas the maximum correlation between any fibrosis stage and time point was 0.23. These results show that differential abundance, in general, is not well conserved between proteins in the cell system and patient samples. This analysis is consistent with our previous analysis [2] showing that there was a subset of proteins displaying similar temporal progression in the cell culture and liver biopsies since the current analysis compared the regulation of all proteins, and did not incorporate the temporal information. We discuss these findings further in the Conclusion section.

We next examined the agreement between topological measures between the cell-culture derived network and the network derived from liver biopsy samples. The Spearman rank correlation comparing betweenness measures in proteins in both networks was 0.4. Though not perfect agreement, this correlation is much better than the correlation obtained comparing differential regulation. To examine this in a slightly different way, we examined the distribution of betweenness values from the clinical network in topological bottlenecks, using a two-sided t-test. We found that bottlenecks in the cell culture network have a significantly higher mean betweenness (from clinical network) than non-bottleneck proteins (138 versus 42, *p*-value 0.02). These results indicate that the general topology of the networks is more conserved than is differential regulation of the individual proteins in each dataset, supporting our notion that network topology provides information not provided by differential regulation in some cases.

**Figure 6:**
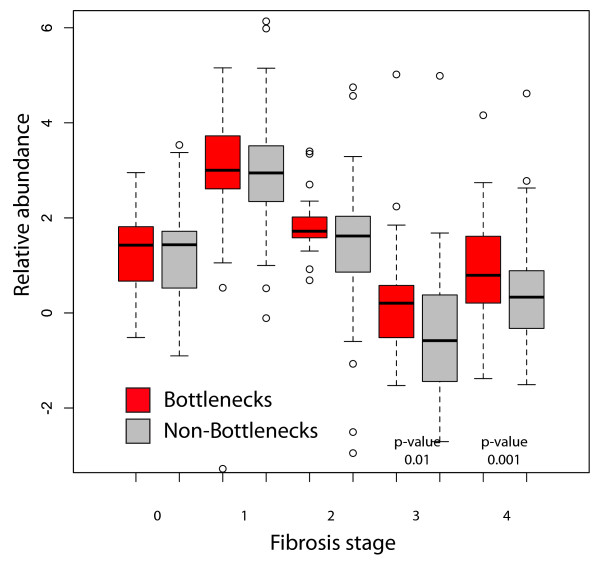
**Bottlenecks from cell culture-derived networks are enriched in proteins differentially regulated in HCV-positive patient samples**. Bottlenecks were identified from a network derived from HCV infection of Huh-7.5 cells. The distribution of differential abundance values in proteomics from patient samples from the bottlenecks (red) versus non-bottlenecks (grey) are shown with the mean indicated by the dark line, the boxes are at 25th and 75^th ^percentiles, and the dashed bars are at 1.5 time the interquartile range. *P*-values indicated are from a two-sided t test.

As we showed, the differential regulation of proteins in both datasets correlates with the probability that these proteins have been identified as pathogen interactors and of HCV in particular. We next examined if the proteins with high betweenness (bottlenecks) in the cell culture network are more likely to be highly differentially regulated in the liver biopsy samples. We first examined the enrichment of bottlenecks in proteins significantly differentially regulated in the clinical samples. This analysis showed that bottlenecks and hubs were both significantly enriched in proteins differentially regulated in liver biopsy samples (*p*-values 0.009 and 0.0009, respectively). We next compared the mean differential regulation of bottlenecks and non-bottlenecks in the patient data in groups separated by stage of fibrosis. We found that bottlenecks were significantly more differentially regulated in patients with late stage fibrosis (3 or 4; see Figure 6), but that this relationship was not observed for hubs (data not shown).

### Network topology provides better target identification than differential regulation

We postulated that network topology could provide better discrimination of interesting proteins than differential regulation. To address this we compared the enrichment of known pathogen interactors, which we consider to be interesting targets, in the most highly differentially regulated proteins from each dataset (see Figure 1) and the proteins with highest betweenness in the network derived from each dataset. This analysis revealed that the top differentially regulated proteins (top 20%) from any time point in the cell culture data set were comprised of 20% known pathogen interactors (relative to 14% in the remaining portion of proteins). The proteins with the top betweenness (bottlenecks; top 20%) from the cell culture-derived network were comprised of 32% known pathogen targets (relative to 10% background). This observation is consistent with results in the liver biopsy data where the maximum enrichment in the top differentially regulated proteins was 17% versus an enrichment of 33% for top ranked bottlenecks. These numbers were also reflected in HCV-specific targets (data not shown). Collectively, these results show that network topology provides better identification of target proteins than does simply ranking by differential regulation.

**Figure 7:**
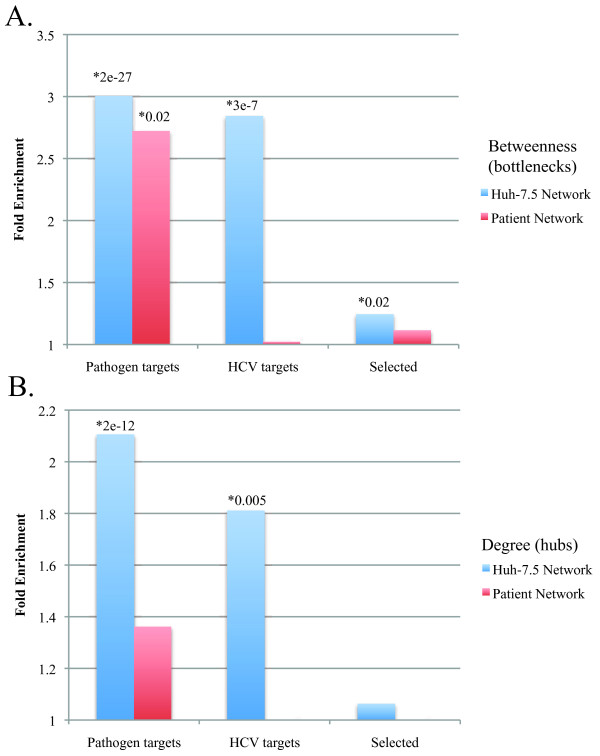
**Topological enrichment of best networks**. Bottlenecks **(A) **or hubs **(B) **were identified in the best networks inferred from the Huh-7.5 (blue bars) or fibrosis patient (red bars) proteomics data. Bottlenecks or hubs were examined for enrichment in general pathogen interactors (pathogen targets), specific HCV interactors (HCV targets), and proteins with cognate genes that exhibit positive evolutionary selection (selected). Fold-enrichment is calculated as the percentage of annotated proteins in the bottleneck group divided by the percentage of annotated proteins in non-bottlenecks or non-hubs as appropriate. Statistical significance was evaluated by Fisher's exact test, *p*-values for significant enrichment are indicated.

Finally, we show a comparison of target identification in both networks using several methods of assessing importance. We compared the enrichment of bottlenecks or hubs in the network inferred from the Huh7.5 cell culture proteomics data with those in the network from the fibrosis patient proteomics data. Figure 7 shows the enrichment of bottlenecks or hubs in each of these networks in general targets of pathogens, HCV-specific targets, and in proteins that exhibit evolutionary positive selection. These results show that bottlenecks (Figure 7A) in both networks are significantly enriched in pathogen interactors, and the bottlenecks in the cell culture network are enriched in specific HCV interactors. It is unclear why the enrichment in HCV-specific interactors is higher in the Huh7.5-derived networks. This difference could be because the processes involved in fibrosis progression *in vivo *involve more than simply HCV replication, and thus bottlenecks in the liver biopsy-derived network may represent proteins with more complicated roles in pathogenesis and thus those less likely to be identified as HCV interactors from the two-hybrid study [11]. Bottlenecks in the cell culture network are also significantly enriched in proteins exhibiting positive evolutionary selection. A similar result was obtained examining hubs in the network (Figure 7B), though the enrichment was significantly less pronounced than in the bottleneck enrichment. This indicates that bottlenecks are more important in both networks, either because they are preferentially interaction targets for pathogens, or that they have been under recent selective pressure.

**Figure 8:**
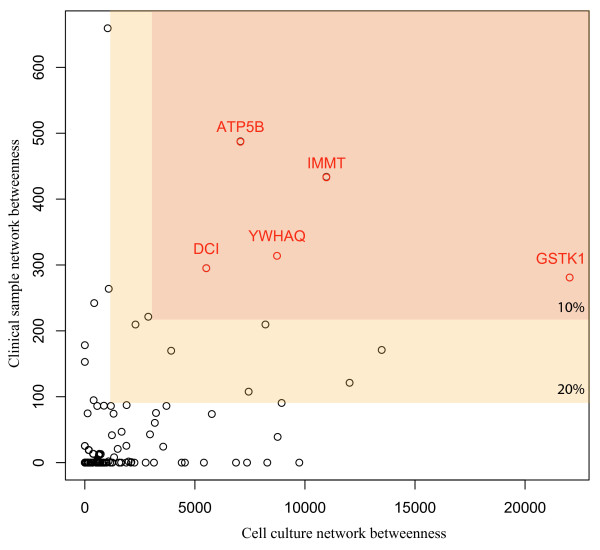
**Overlap of predicted bottlenecks in networks from cell culture and patient data**. Bottlenecks (top 10% or 20% as indicated) were identified for proteins in the networks derived from the HCV infected Huh-7.5 cell proteomic data and from liver biopsy samples from HCV-positive patients. Proteins (points) are plotted based on their betweenness centrality (number of paths passing through them) from the cell culture network (X axis) versus in the clinical network (Y axis). Boxes indicate a 10% or 20% threshold for bottleneck identification in both networks. Protein names for high-confidence shared bottlenecks are indicated.

Given the importance of bottlenecks in both networks we were interested in determining which of these proteins represented conserved bottlenecks in both networks. We postulate that these common bottlenecks will be even more important to HCV replication and pathogenesis. Figure 8 shows a plot of the betweenness values from each network for each protein with proteins identified as high-confidence (10%) bottlenecks in both networks highlighted in red. This small group includes ATP5B, DCI, GSTK1, IMMT, and YWHAQ. Interestingly, several of these proteins are localized on the mitochondria including DCI, a mitochondrial fatty acid oxidation enzyme whose requirement in the HCV life cycle we have now confirmed by a series of *in vitro *perturbations including gene silencing and pharmacologic inhibition [7]. All shared bottlenecks are listed in Table 2 and a complete list of all the shared proteins from the two networks is provided as (Additional file 7: Table S5). Finally, functional enrichment of the shared bottleneck proteins shows that this groups is significantly enriched in proteins localized to mitochondria and having mitochondrial-related functions (Benjamini-Hochberg adjusted *p*-value 1.0e-6).

**Table 2:** Conserved bottlenecks between cell culture and clinical samples

**Symbol**^**a**^	ID	Description	**Notes**^**b**^
GSTK1	NM_015917	glutathione S-transferase kappa 1	M

IMMT	NM_006839	inner membrane protein, mitochondrial (mitofilin)	P, M

DCI	NM_001919	dodecenoyl-Coenzyme A delta isomerase	M

ATP5B	NM_001686	ATP synthase, H + transporting	M

YWHAQ	NM_006826	tyrosine 3-monooxygenase/tryptophan 5-monooxygenase activation	P

HSPA8	NM_006597	heat shock 70 kDa protein 8	P, E

CALR	NM_004343	calreticulin	P, H, E

RPLP1	NM_001003	ribosomal protein, large, P1	

CYCS	NM_018947	cytochrome c, somatic	P, M

ETFA	NM_000126	electron-transfer-flavoprotein, alpha	M

HSPA9	NM_004134	heat shock 70 kDa protein 9 (mortalin)	P

GOT2	NM_002080	glutamic-oxaloacetic transaminase 2	M, E

^a^Genes appearing in the top 20% of bottlenecks ranked in both cell culture and clinical networks. These are also shown in the yellow and orange shaded regions of Figure 8

^b^P, pathogen interactor; H, HCV target; M, mitochondrial protein; E, positive evolutionary selection

To further investigate potential functional roles of these conserved bottleneck proteins we performed functional enrichment on the first-order networks of each protein. The first-order networks for several conserved bottlenecks are shown in (Additional file 8: Figure S2) and the functional categories significantly enriched in each neighborhood are listed in (Additional file 9: Table S6). Though many of the individual conserved bottleneck proteins were linked to mitochondria (Table 2), the functions of their neighborhoods are fairly diverse. However, two neighborhood networks were significantly enriched in processes related to fatty acid metabolism and its regulation (DCI and YWHAQ). Additionally, we provide the topology of the conserved targets in both networks in (Additional file 10: Table S7). These results show that many of the bottlenecks are also hubs (highly connected proteins) in both networks including CALR, ETFA, IMMT, and RPLP1. Interestingly, all of the targets have low clustering coefficients. The clustering coefficient is the percentage of a node's neighbors that are also linked to each other and reflects the density of edges in that portion of the network. Given that betweenness is a primary driver of importance in the network (Figure 4B) this is not a surprising observation. That is, even hubs having many neighbors may be playing connecting roles in the network because they are also bottlenecks, and a high density of edges in their neighborhoods would decrease their betweenness since this would provide multiple routes through their neighborhood.

## Conclusions

We previously described network analyses of cell culture data to define interactions between host and pathogen and identified mitochondrial fatty acid oxidation enzymes that are predicted to function as central points for connecting and controlling metabolic pathways and as such, key targets in HCV-associated metabolic reprogramming [2]. In fact, dys-regulations in mitochondrial function are evidenced by wide-spread perturbation of related proteins across every HCV model system we have studied [2,5,7,26,27]. Thus, the modeling efforts reported here leveraged these data to further investigate whether the parameters used in our prior *in vitro *modeling activities (abundance filter of 0, correspondence filter of 4, and correlation filter of 0.9) were the best to help identify new targets. Indeed, our previous study did not examine whether the use of a network topology approach could identify important targets any better than a standard approach such as considering highly differentially regulated proteins. Additionally, though we previously showed that this approach was valuable in cell culture studies it also remained unclear how it would perform on data from very different kinds of samples, such as those from liver biopsies of HCV-positive patients.

In the current study we build upon our previous findings to determine if there are other topological metrics (for example clustering coefficient and closeness) that identify targets in these networks, to define the parameters for network construction that provide the best target identification, and to characterize the relationship between networks derived from the cell culture data and those derived from data from patient biopsies. Our results indicate that the method of network construction has a significant impact on the results obtained. We found that betweenness was the most effective metric for defining important targets in our network but that other topological metrics (degree, clustering coefficient and closeness) could also discriminate targets to a statistically significant extent. From previous work examining the properties of topological bottlenecks in networks inferred from global transcriptomics data we have postulated that bottlenecks may represent mediators of transitions between states of the system [1,28-30], and therefore represent critical points of control for the disease process. We have speculated that this is because bottlenecks link functional modules that represent groups of genes or proteins coexpressed under similar conditions. The transition between modules may represent state changes in the system, and the position of bottlenecks makes them candidates for regulators of these transitions. Our finding that degree was also a good predictor of importance in the system reiterates previous findings in other undirected biological networks [23], though the primary contribution to importance we found to be betweenness, similar to findings in regulatory networks. Our findings here are consistent with the idea that bottlenecks in coabundance networks represent transitions between functional modules, and show that bottlenecks and hubs from proteomics-based networks may have similar properties as those from transcriptomics-based networks.

We note that modeling activities involving integrated genomic-proteomic analyses is an important area of research aimed at understanding the differences between co-expression at the transcript and protein level. However, our initial modeling efforts centered on the utilization of proteomic and metabolomic data indicating a temporal regulation of cellular metabolic homeostasis that was not detected by the accompanying gene expression profiles. Indeed our prior *in vitro *studies were unique in part because they described a previously un-identified role for post-transcriptional regulatory mechanisms in the metabolic rerouting that was observed [2]. For this reason, the scope of the current manuscript has focused on extending our analyses specifically to comparison with *in vivo *protein co-expression networks.

Upon optimization of network construction, subsequent comparative analyses revealed that topologically-defined bottleneck proteins in the cell culture-derived network were generally more differentially regulated in patients with advanced fibrosis than their non-bottleneck counterparts. Interestingly, this was not observed when comparing differential abundance alone between the two datasets, indicating that topological analysis may identify more clinically relevant targets from cell culture studies than relative expression. It is important to note that we previously identified a subset of proteins that showed strongly conserved patterns of differential abundance [2] between the cell culture and liver biopsy samples. In the current analysis we show that as an overall measure, differential abundance does not correlate well between the two data sets. Additionally, bottlenecks in the cell culture network were more likely to be bottlenecks in the clinical network. This shows that our approach can identify proteins of interest based on cell culture studies that are important in human disease and that these proteins would not be identified by examining differential abundance alone. Importantly, these findings point to the limitations of identifying/prioritizing pathogen-host targets based solely on highly differential regulation, a common approach to the identification of targets for further investigation.

Throughout this study we refer to target proteins as proteins that are important for HCV replication and/or fibrosis development. Some of these proteins have been defined using two-hybrid screens [11,21], but our working hypothesis is that there are proteins that are important for replication that have not been previously defined. These are proteins that may or may not be direct interaction partners with HCV proteins but could contribute to metabolic or signaling pathways necessary for HCV replication and/or liver disease progression. We previously proposed an important role for temporal regulation of mitochondrial fatty acid oxidation and energy production in HCV infection and liver disease progression. Briefly, we described early increases in mitochondrial fatty acid oxidation that contribute to the creation of a "pro-viral" environment immediately preceding the subsequent increase in viral replication observed *in vitro *[2]. This was eventually followed by a decline in fatty acid oxidation that accompanied the appearance of a cytopathic effect *in vitro *and liver disease progression *in vivo *[2]. The down-regulation of mitochondrial fatty acid oxidation would favor an increase in hepatocellular lipid content (for example, steatosis), a common occurrence in HCV, and histological feature observed among 4 of 6 patients with advanced fibrosis in our *in vivo *studies [5]. The conservation of protein abundance changes associated with pathogenesis *in vitro *(e.g. cytopathic effect) and liver disease progression *in vivo*, and the corresponding mitochondrial bottlenecks reported here, including DCI, raises the interesting prospect that these proteins play an important role in the viral life cycle and pathogenesis.

Our previous findings and those described in the current study prompted us to further explore the predicted influence of HCV-associated disruptions in mitochondrial fatty acid oxidation, including consideration of whether these perturbations would be reflected by disease-related patterns detected in blood. From a clinical perspective, biomarker discovery efforts in body fluids represent an attractive alternative to tissue samples owing to the relative ease and less invasive nature of collection and the large volumes that normally can be obtained. We have observed the accumulation of both substrates for enoyl-CoA isomerase activity (e.g. DCI) as well as dicarboxylic acids well known to reflect alternative fatty acid catabolism through ω-oxidation pathways, findings consistent with our predictions regarding an important role for DCI, the essential link between saturated and unsaturated β-oxidation, in the impaired mitochondrial fatty acid catabolism occurring during HCV-associated liver disease progression [28]. Thus, the identification of disease-related fatty acid patterns in the blood of patients with HCV-associated liver disease progression provides a potentially useful noninvasive diagnostic link to the previously described alterations in hepatic mitochondrial fatty acid oxidation occurring during HCV infection and pathogenesis. Importantly, we have unequivocally validated a biologically relevant role for DCI in the HCV life cycle using a combination of gene silencing and pharmacologic inhibition approaches [2,5,7]. In summary, our data from multiple model systems and clinically relevant physiologic compartments provide evidence confirming our original modeling predictions regarding a requirement for DCI in the HCV life cycle [7] and demonstrate a physiologically relevant association of temporal declines in fatty acid oxidation that coincide with pathogenesis *in vitro *and *in vivo*. Taken together, we believe these data provide proof of principle for the utility of integrated *in vitro/in vivo *modeling efforts to identify key host targets of HCV infection and pathogenesis.

The biological interpretation of the remaining top 10% bottlenecks, 4 out of 5 of which are mitochondrial proteins with links to fatty acid oxidation and energy production, was predicated on the wealth of data described for the representative example DCI as highlighted above together with the growing literature on the important role of altered mitochondrial function in HCV infection and pathogenesis (for an excellent review on the interactions between HCV and mitochondria we recommend [29]). Among the additional bottlenecks identified was glutathione-S-transferase kappa 1 (GSTK1), a protein that localizes to the mitochondria and peroxisome and has pleiotropic functions including glutathione conjugation, peroxidase, and disulphide-bond-forming oxidoreductase activities [30]. Interestingly, GSTK1 has recently been shown to play an important role in the oligomeric assembly and secretion of adiponectin, a cytokine that stimulates fatty acid oxidation through interaction with the hepatic receptor AdipoR2 and subsequent activation of peroxisome proliferator-activated receptor (PPAR)-alpha [31,32]. HCV-associated targeting of GSTK1 and DCI may serve to provide multiple control points for modulating catabolic flux of fatty acids during metabolic reprogramming. GSTK1 may promote further cross-talk between metabolic signaling and biochemical pathways by modulating the folding and assembly of oligomeric proteins directly involved in lipid synthesis and/or catabolism, including the trimeric DCI protein. A similar role in the folding of lipid metabolism enzymes has been suggested in *Caenorhabditis elegans *where GSTK1 silencing was associated with a decline in the biosynthesis of the monounsaturated fatty acid *cis*-vaccenic acid [33]. It is worth noting that the differential abundance of *cis*-vaccenic acid was observed to impact lipid droplet remodeling under pathogenic conditions of defective peroxisomal β-oxidation in *C. elegans *[34]. Taken together, these findings suggest interesting new avenues of research aimed at exploring the interplay between GSTK1 and DCI during metabolic reprogramming and the lipid remodeling events predicted to provide important constituents in the various structural entities supporting the HCV life cycle, including the lipid droplet and membranous replicase compartments.

Among the other bottlenecks detected in our analyses was mitofilin, also known as mitochondrial inner membrane protein (IMMT). Mitofilin is a protein localized to the inner mitochondrial membrane whose presence is essential for tubular cristae formation and the increased surface-to-volume ratio of the inner membrane that occurs during increased metabolic output [35]. While the molecular basis for these alterations in mitochondrial cristae morphology are not well understood, mitofilin depletion has been shown to induce aberrant structural changes in the inner membrane that are associated with abrogation of ATP production despite increased flux of fatty acid substrates through the β-oxidation pathway thus, suggesting an adverse impact on the oxidative phosphorylation machinery that resides in the inner membrane [35]. We suspect that the putative HCV targeting of mitofilin reflects a coordinated effort to maximize energy production in support of the significant macromolecular biosynthesis necessary for viral growth [2]. Consistent with this idea we further identified ATP5B, the major catalytic subunit of F1 ATP synthase, as a conserved bottleneck in our studies. A similarly important pro-viral role for ATP5B has recently been reported for herpes simplex virus-1 (HSV-1) [36]. In a series of elegant experiments aimed at exploring the effect of host microRNAs on HSV-1 replication, Zheng *et al*, identified a point of cross talk between host cell and virus that results in the progressive induction of host cell miR-101 levels that is accompanied by concomitant declines in ATP5B expression and HSV-1 replication [36]. The interplay between virus and the miR-101/ATP5B regulatory network suggests a potential link between modulation of this host defense mechanism and the establishment of long-term HSV-1 latency [36]. This latter point is of particular interest as we and others have proposed a similar role for modulation of fatty acid oxidation and energy production in the establishment of persistent HCV and measles virus infection [2,37].

It is important to note that our intent is not to provide a network representation that is faithful to the underlying true network of interactions in the cell, but rather to use topology in these simply defined association networks to identify target proteins for further experimental investigation. The networks generated using this approach are based on correlation of protein abundance over many different observations (time points in the cell culture data and patients in the clinical data). As such they represent the information flow in the system. For example, closely coordinated proteins are close together in the networks, while those with little or no coordination are far apart. It is likely that this organization allows use of topology to query the network for more important proteins, since bottlenecks in particular represent points constriction in information flow in the system [23]. In a fashion analogous to that for DCI, additional conserved bottleneck proteins represent particularly attractive targets for further investigation of their functional significance during HCV infection and liver disease progression. In this regard, recent efforts to link these findings with clinical protein profiling studies of serial liver biopsies obtained from HCV-positive liver transplant recipients revealed a statistically significant up-regulation of the protein bottleneck GSTK1 in patients who developed severe liver injury [28]. Importantly, the increased abundance of GSTK1 occurred prior to histologic evidence of fibrosis. Collectively, these findings merit further investigation to understand the functional, regulatory and/or prognostic significance of this protein bottleneck during HCV-associated liver disease progression.

In summary, the results presented in this study show that a network approach to consideration of global proteomics data is a powerful way to identify important target proteins and to elucidate potential mechanisms of pathogenesis. Previous results in yeast [23,38], fruit fly and worm [39], pathogenic bacteria [40,41], cyanobacteria [42], mouse macrophages [43], mouse blood [44] and human cell culture [2,7] support the notion that our approach is generally applicable, though these have been focused on analysis of coexpression networks from transcriptomics. We have recently published on the network analysis of proteomics data from *Salmonella *under infectious-like conditions, and have found that these networks show a similar kind of enrichment of bottlenecks in proteins important to the system [45]. In the current work we fully characterize the application of this approach to protein co-abundance networks showing that it works very well to identify important nodes in the network. In this study we show that topological betweenness provides the best identification of important target proteins, but that other topological measures can also be used to identify targets. Importantly, we show that this approach can be applied successfully to global proteomic data derived from liver biopsies of HCV-positive fibrosis patients. Key findings of the study were validated in a patient cohort by metabolic profiling in serum [28]. Interestingly, the topology of cell culture networks provides better insight into important proteins in the liver biopsy data than does differential regulation, showing that it is a viable alternative or complement to standard analysis methods. Our approach represents a generally applicable method for using global proteomics data as a systems biology tool that goes beyond differential abundance of individual proteins. The finding that other metrics could also identify targets suggests that combining network metrics in some way may provide improved discrimination over the individual measures. Our initial results using a simple mean, geometric mean, or minimum of protein rank from each of the four metrics revealed that the results were not improved (data not shown). We are currently investigating more sophisticated methods for integrating multiple topological measures to improve our results.

## Competing interests

The authors declare that they have no competing interests.

## Authors' contributions

JEM: conceived the methods, developed code, performed the analysis, wrote the paper. DLD: provided biological interpretation, wrote the paper. CC: developed code and performed analysis. ALR: provided biological interpretation. MGK: provided funding for the work performed, provided biological interpretation. KMW: conceived the methods, provided biological interpretation. All authors read and approved the final manuscript.

## Supplementary Material

Additional file 1 **R scripts used for network inference, topological analysis and functional enrichment**. Click here for file

Additional file 2 **Table S1**. Enrichment of abundant proteins in pathogen interactors. Click here for file

Additional file 3 **Table S2**. Topological analysis of networks inferred from cell culture proteomics data using different parameters. Click here for file

Additional file 4 **Figure S1**. Correlation between topological properties in four types of networks. Click here for file

Additional file 5 **Table S3 Topological analysis of networks inferred from liver biopsy proteomics data using different parameters**. Click here for file

Additional file 6 **Table S4**. Summary enrichment of networks generated from liver biopsy proteomics data using different parameters. Click here for file

Additional file 7 **Table S5**. Complete list of shared proteins from cell culture and patient derived networks. Click here for file

Additional file 8 **Figure S2**. First-order networks of conserved bottleneck proteins. Circles represent proteins with shared bottlenecks colored yellow, lines represent correlation relationships or protein-protein interactions between proteins. **A**. ATP5B. **B**. DCI and IMMT. **C**. GSTK1. **D**. HSPA8. **E**. YWHAQ. Click here for file

Additional file 9 **Table S6**. Functional enrichment of shared bottleneck neighborhoods. Click here for file

Additional file 10 **Table S7**. Network topology of conserved bottlenecks in cell culture and patient derived networks. Click here for file
