# Supplementary material for: Topological analysis of protein co-abundance networks identifies novel host targets important for HCV infection and pathogenesis
Source: BMC Syst Biol. 2012 Apr 30;6:28. doi: 10.1186/1752-0509-6-28 (PMC3383540; doi:10.1186/1752-0509-6-28)
Supplement: Additional file 4 — Figure S1. Correlation between topological properties in four types of networks. [file 1752-0509-6-28-S4.PDF]

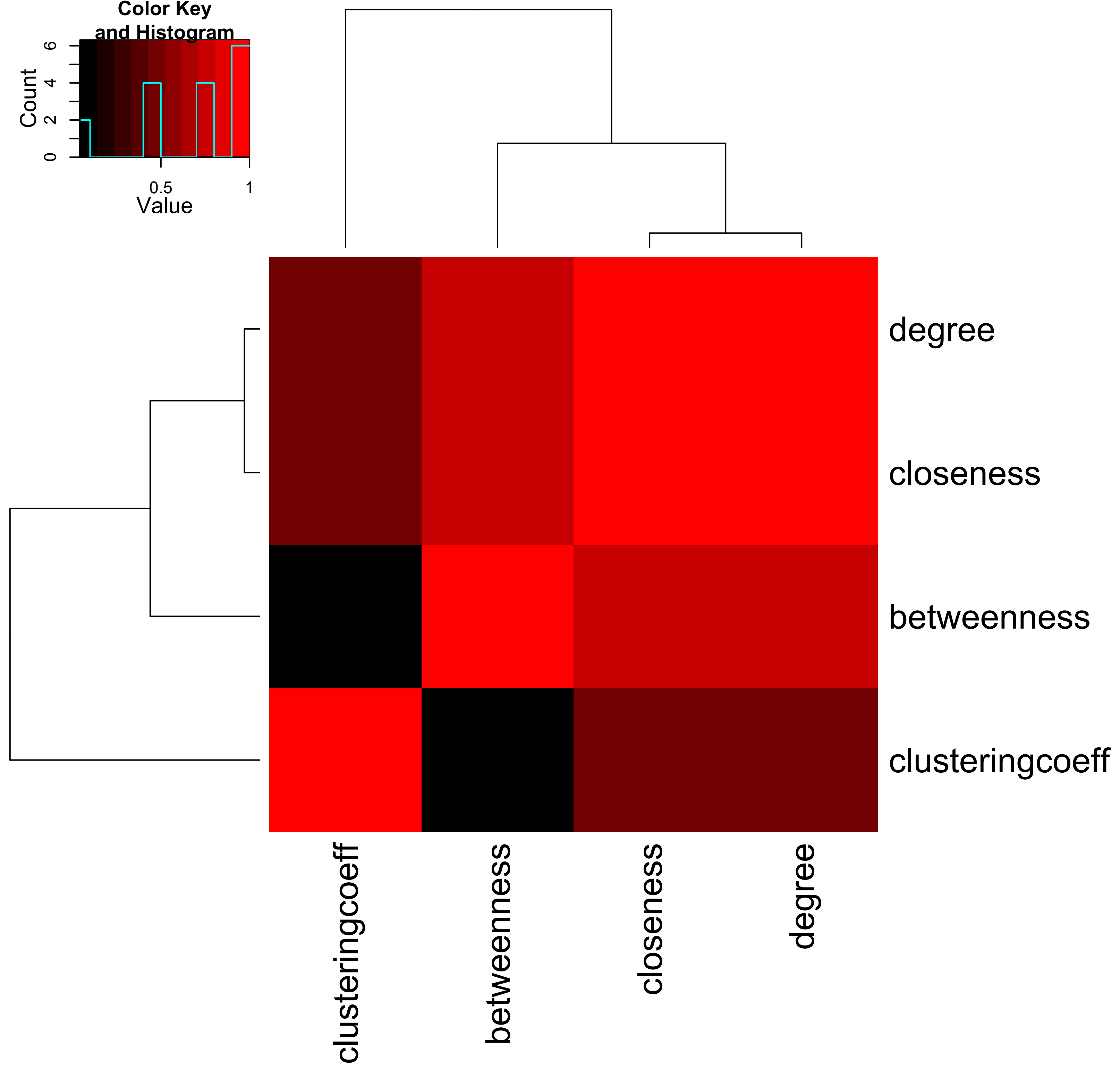

A. Cell culture co-abundance

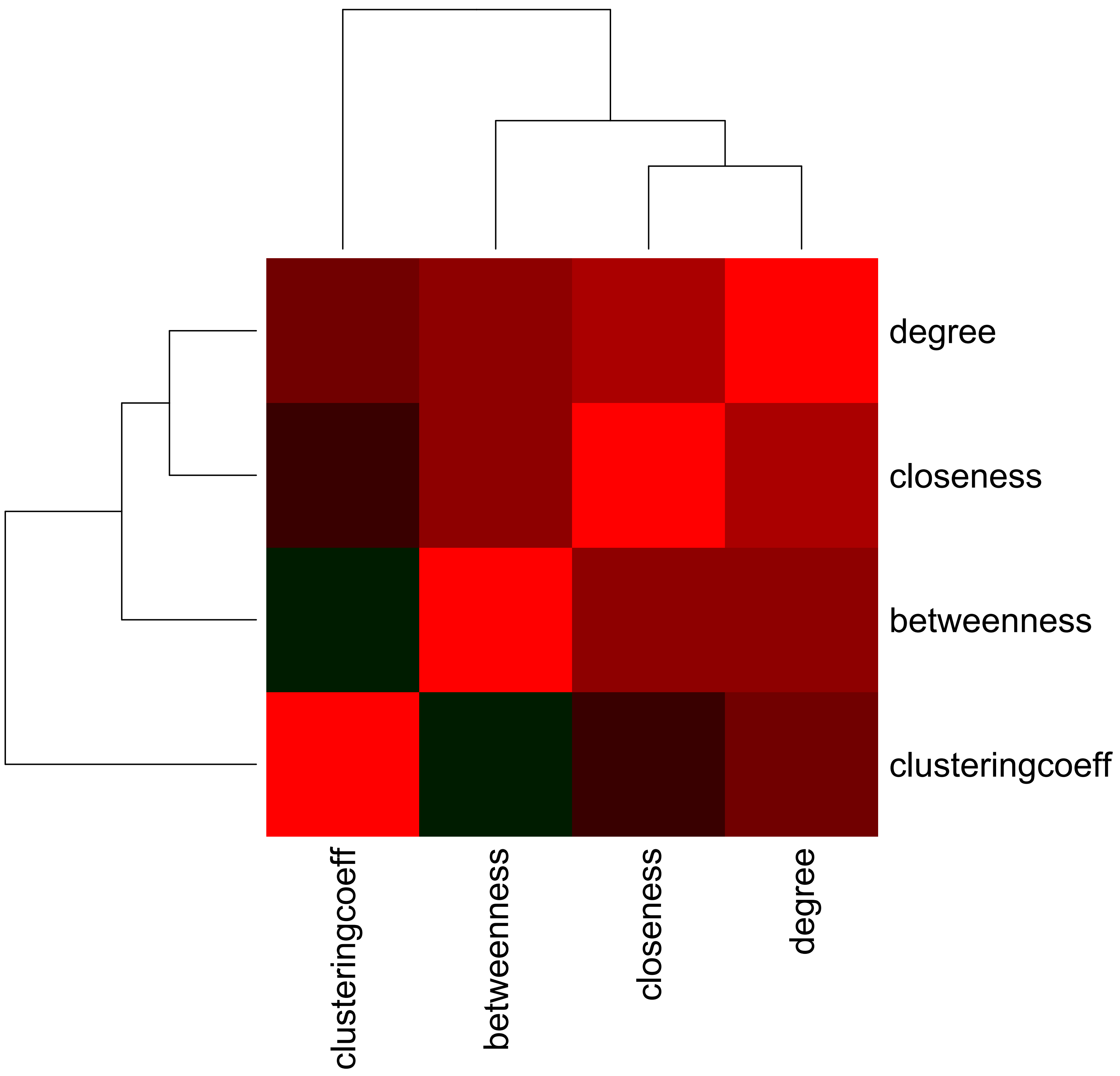

B. Cell culture co-abundance + PPIs

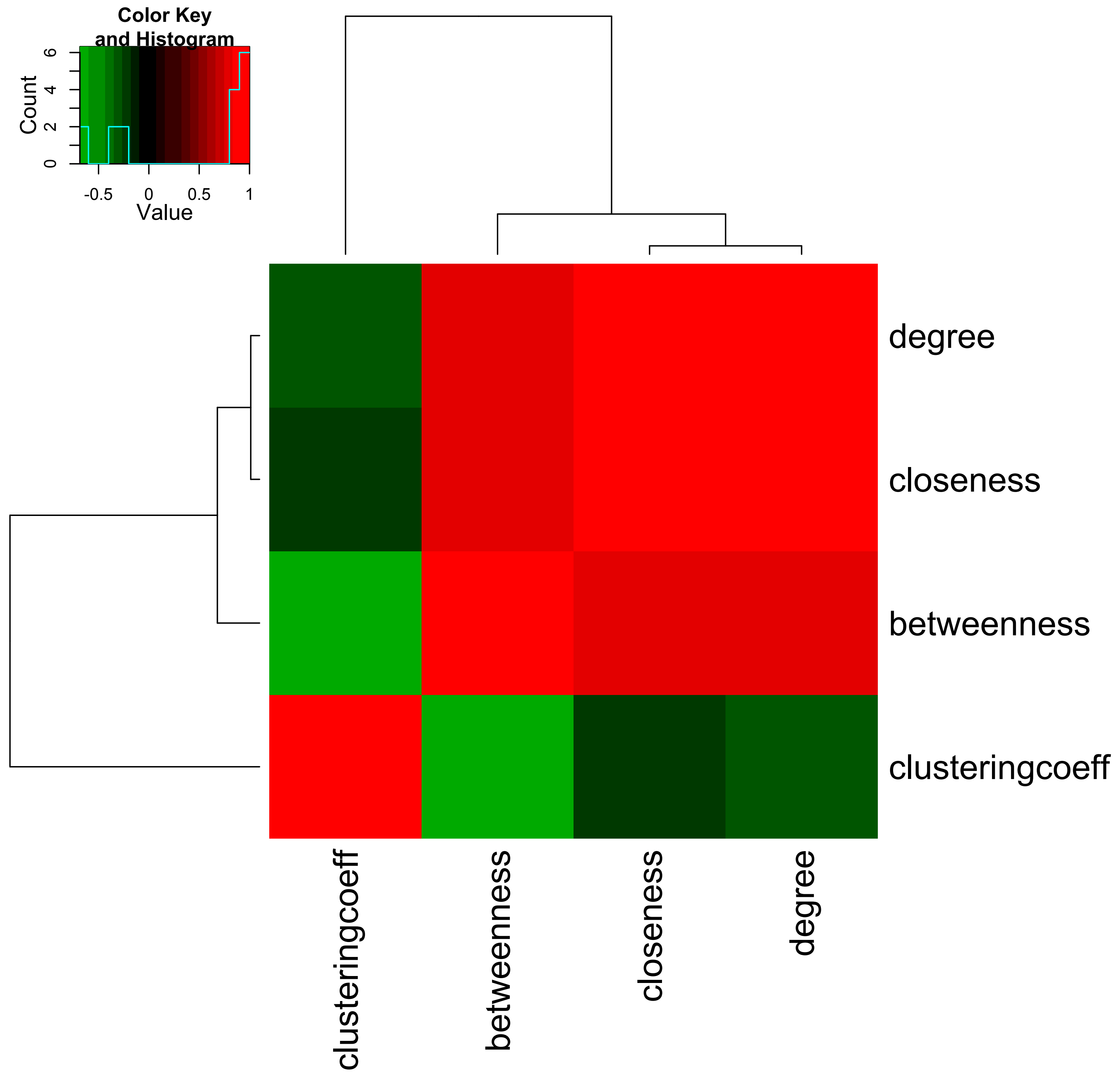

C. Clinical co-abundance

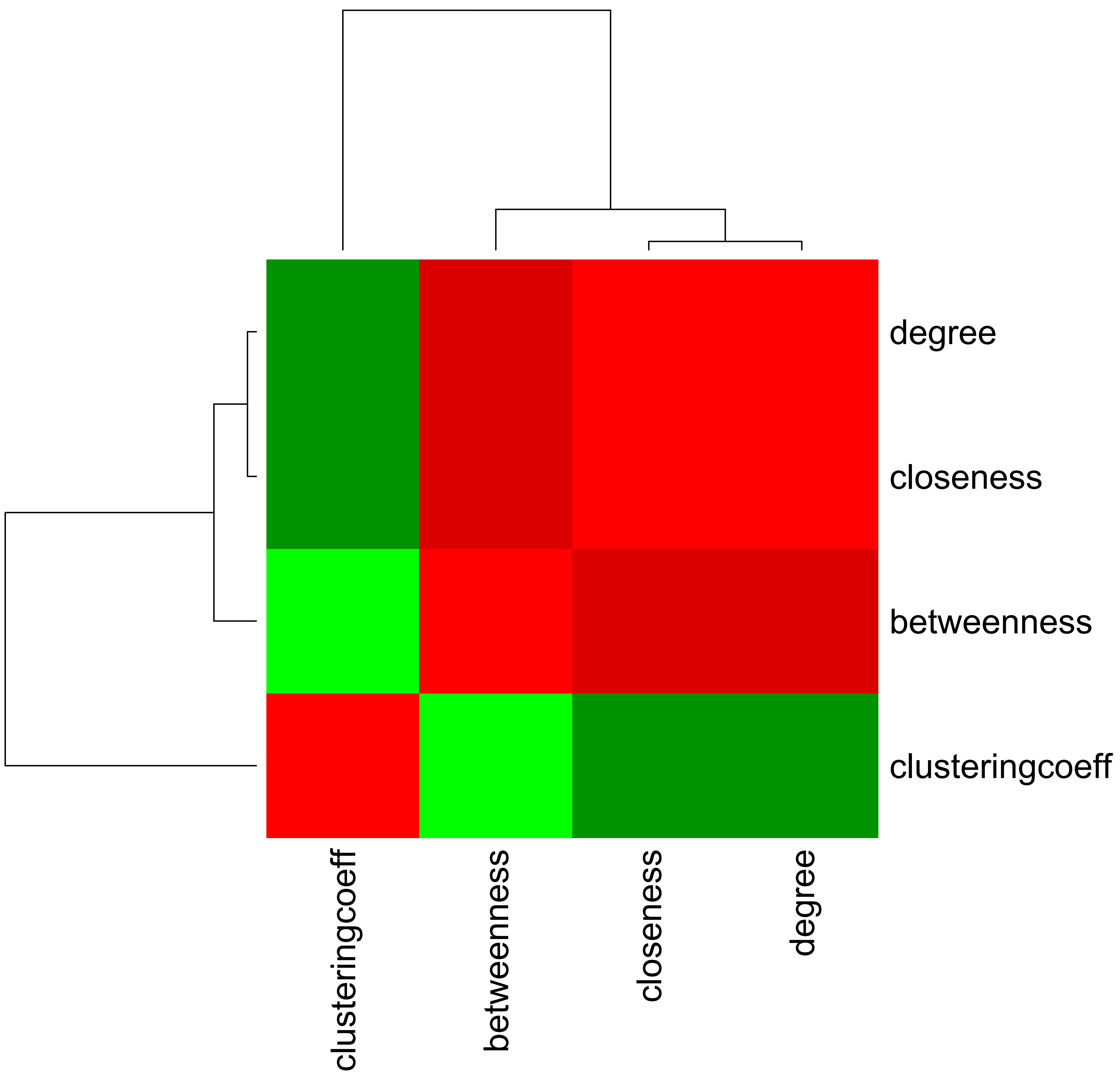

D. Clinical co-abundance + PPIs

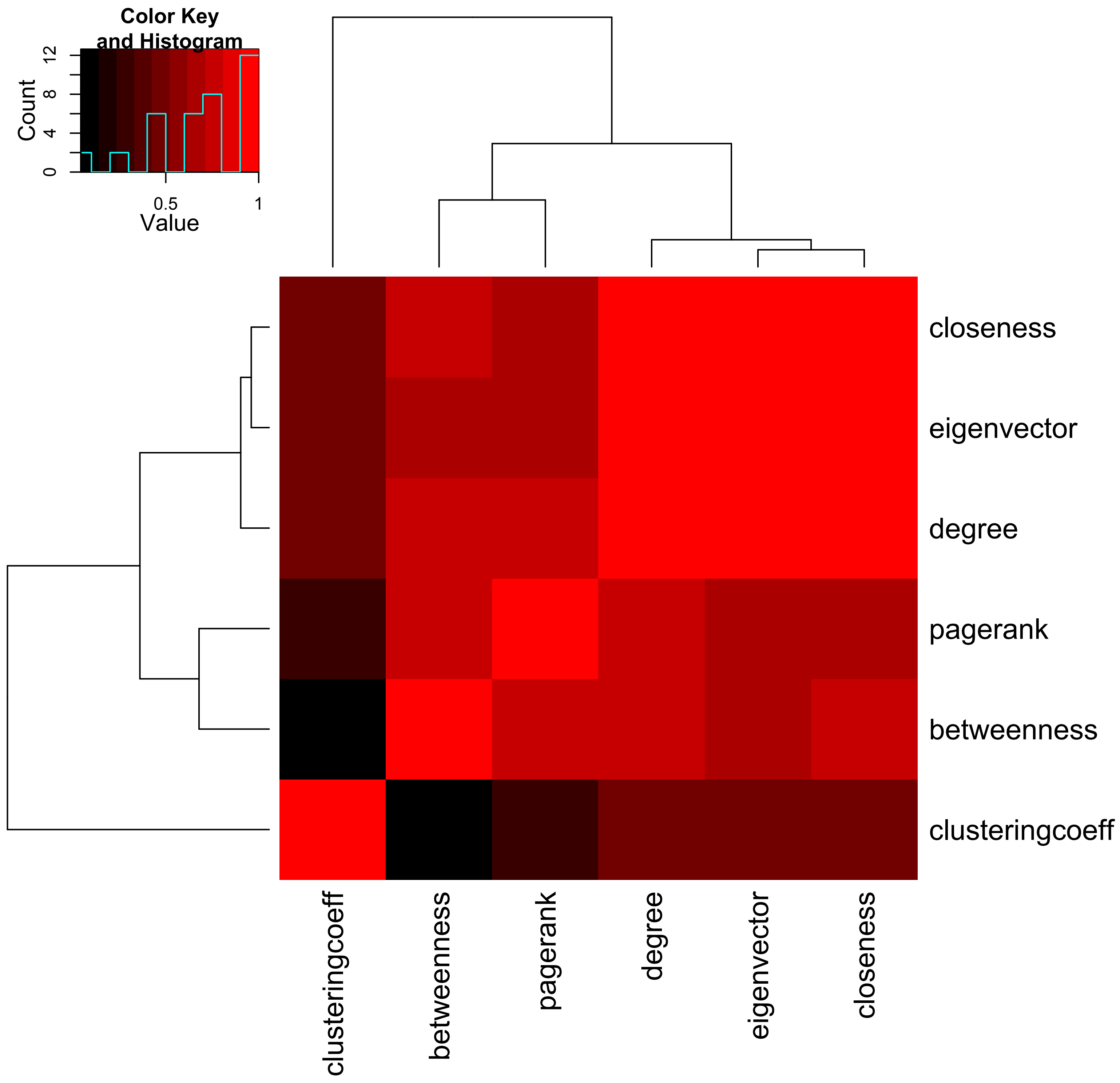

E. Cell co-abundance w/all topology measures
