# Supplementary material for: Topological analysis of protein co-abundance networks identifies novel host targets important for HCV infection and pathogenesis
Source: BMC Syst Biol. 2012 Apr 30;6:28. doi: 10.1186/1752-0509-6-28 (PMC3383540; doi:10.1186/1752-0509-6-28)
Supplement: Additional file 8 — Figure S2. First-order networks of conserved bottleneck proteins. Circles represent proteins with shared bottlenecks colored yellow, lines represent correlation relationships or protein-protein interactions between proteins. A. ATP5B. B. DCI and IMMT. C. GSTK1. D. HSPA8. E. YWHAQ. [file 1752-0509-6-28-S8.PDF]

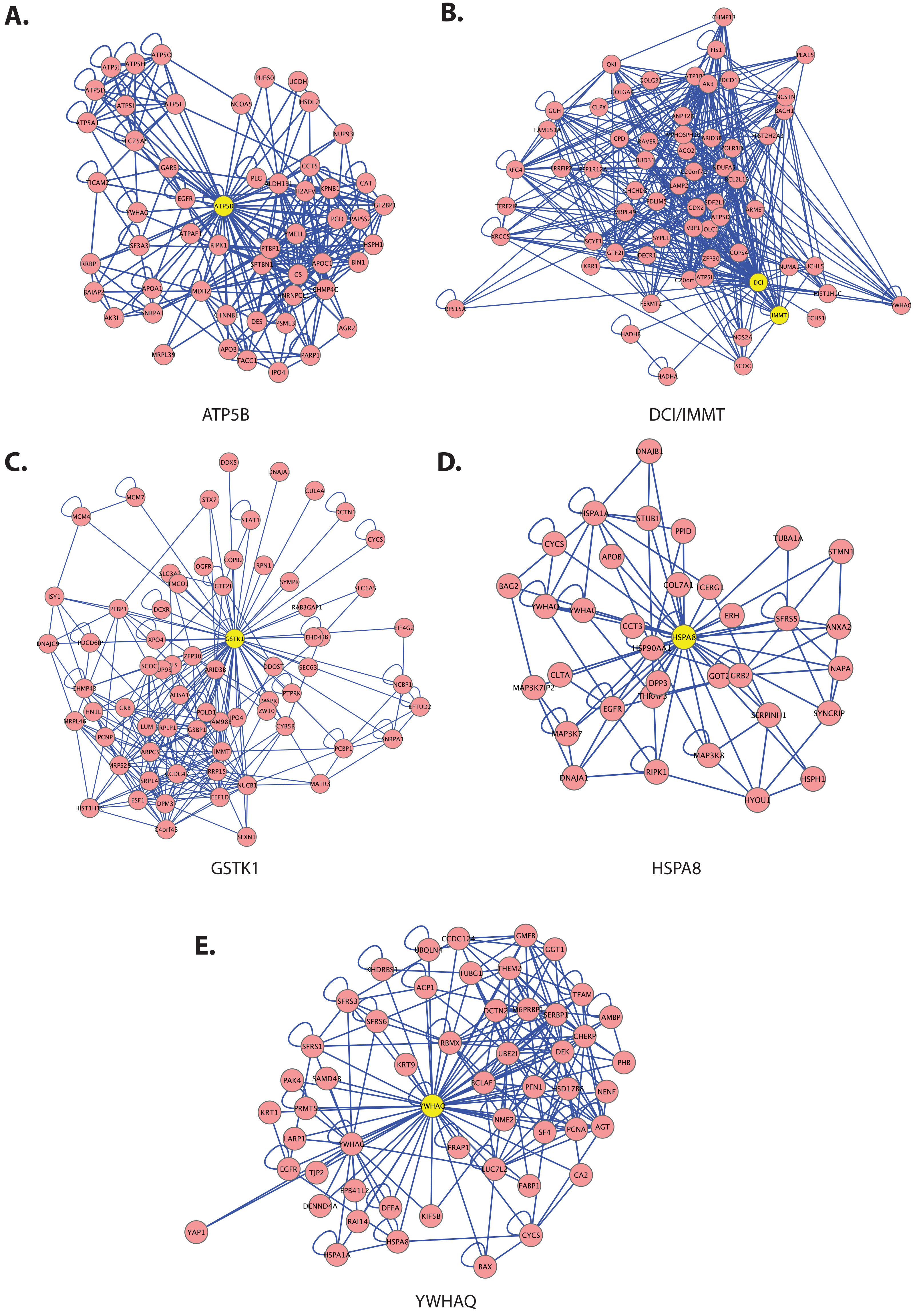

A.
ATP5O
ATP5H
ATP5J
ATP5D
ATP5I
ATP5F1
ATP5A1
SLC25A5
PUF60
UGDH
NCOA5
HSDL2
NUP93
GARS
TICAM2
EGFR
ATP5B
PLG
ALDH1B1
CCT5
H2AFV
KPNB1
CAT
IGF2BP1
YWHAG
PGD
PAPSS2
ATPAF1
SF3A3
RIPK1
PTBP1
YME1L1
HSPH1
RRBP1
SPTBN1
APOC1
BIN1
CS
APOA1
BAIAP2
MDH2
HNRNPCL1
CHMP4C
AK3L1
SNRPA1
CTNNB1
DES
PSME3
AGR2
MRPL39
APOB
TACC1
PARP1
IPO4
ATP5B
B.
CHMP1B
FIS1
PEA15
QKI
GOLGB1
ATP1B
AK3
PDCD1
NCSTN
GOLGA
GGH
CLPX
BACH1
FAM151A
CPD
RAVER1
ARID3B
HIST2H2AB
POLR1D
RFC4
BUD31
NDUFA5
C20orf72
LAMP2
BCL2L15
CHCHD
PDLIM5
CDX2
SDF2L1
TERF2IP
MRPL4
ATP5D
ARMET
XRCC5
SCYE1
SYPL1
VBP1
GTF2I
DECR1
ZFP30
COPS4
KRR1
ATP5I
DCI
NUMA1
UCHL5
HIST1H1C
RPS15A
FERMT2
IMMT
ECHS1
YWHAG
HADHB
NOS2A
SCOC
HADHA
DCI/IMMT
C.
DDX5
DNAJA1
CUL4A
MCM7
STX7
STAT1
DCTN1
MCM4
CYCS
COPB2
OGFR
RPN1
GTF2I
SYMPK
SLC1A5
ISY1
PEBP1
DCXR
RAB3GAP1
EIF4G2
DNAJC9
PDCD6IP
XPO4
GSTK1
EHD4
ZFP30
SCOC
ARID3B
DDOST
SEC63
CHMP4B
AHSA1
PTPRK
NCBP1
EFTUD2
HN1L
CKB
POLD1
ZW10
IPO4
MRPL46
LUM
RPLP1
G3BP1
CYB5B
PCNP
ARPC5
IMMT
PCBP1
SNRPA1
MRPS28
CCDC47
RRP15
SRP14
NUCB1
MATR3
ESF1
DPM3
EEF1D
HIST1H1C
C4orf43
SFXN1
GSTK1
D.
DNAJB1
HSPA1A
STUB1
PPID
CYCS
APOB
TUBA1A
STMN1
BAG2
COL7A1
TCERG1
YWHAQ
YWHAG
ERH
CCT3
HSPA8
SFRS5
ANXA2
HSP90AA1
CLTA
DPP3
GOT2
GRB2
NAPA
MAP3K7IP2
EGFR
SERPINH1
SYNCRIP
MAP3K7
MAP3K8
DNAJA1
RIPK1
HSPH1
HYOU1
HSPA8
E.
CCDC124
GMFB
UBQLN4
GGT1
KHDRBS1
TUBG1
THEM2
ACP1
TFAM
SFRS3
DCTN2
M6PRBP1
SERBP1
AMBP
SFRS6
CHERP
SFRS1
RBMX
DEK
UBE2I
PHB
KRT9
PAK4
SAMD4B
BCLAF1
PFN1
HSD17B8
NENF
KRT1
PRMT5
YWHAQ
NME2
SF4
PCNA
AGT
LARP1
YWHAG
FRAP1
EGFR
TJP2
LUC7L2
CA2
FABP1
EPB41L2
DENND4A
DFFA
KIF5B
YAP1
RAI14
HSPA8
CYCS
HSPA1A
BAX
YWHAQ
